# Rebuttal to Correspondence on “Pesticide Residues in Organic and Conventional Agricultural Soils across Europe: Measured and Predicted Concentrations”

**DOI:** 10.1021/acs.est.5c05656

**Published:** 2025-07-02

**Authors:** Dennis Knuth, Vera Silva, Paula Harkes, Jakub Hofman, Violette Geissen

**Affiliations:** † Soil Physics and Land Management Group, 4508 Wageningen University and Research, Droevendaalsesteeg 3, 6708 PB Wageningen, The Netherlands; ‡ RECETOX, Faculty of Science, 37748 Masaryk University, Kamenice 753/5, 625 00 Brno, Czech Republic

In his Correspondence^1^ to our research article “Pesticide Residues in Organic and Conventional Agricultural Soils across Europe: Measured and Predicted Concentrations”,^2^ the author challenges our interpretation of the origin of certain pesticide concentrations of our monitoring study. For this, Benzing analyzes the data provided by us in a targeted way, which was not in the scope of our approach to the data.

We appreciate the engagement with our work and his detailed analysis of pesticide residues in organic soils. His observation that some residue patterns may point to potential cases of noncompliance by organic farmers is a relevant and important point of discussion.

However, we would like to clarify that our study did not aim to examine pesticide-specific differences between organic and conventional farms or to identify potential violations of organic standards. As such, the type of analysis Dr. Benzing conducted was beyond the scope of our exploration of the monitoring data set. The primary objective of our study was to compare measured environmental concentrations with predicted environmental concentrations across Europe, and not compliance monitoring. Therefore, we would not characterize our interpretation of the data set as a misinterpretation, but rather as a focus on a different aspect of the data.

While we acknowledge the presence of certain residue profiles that may appear suspicious, our study relied on voluntary participation and involved extensive sampling across multiple environmental compartments. Under these circumstances, and in the absence of direct evidence, we believe it would be inappropriate and scientifically unfounded to infer fraudulent behavior.

We thank Dr. Benzing for his contribution. His analysis underscores the importance of careful communication of scientific findings, especially in the context of organic certification and public perception. We agree that additional research, specifically targeting potential sources of contamination or noncompliance, would be a valuable complement to our work.
